# A Rare Phenotype of Inherited Cerebellar Ataxia

**DOI:** 10.7759/cureus.28831

**Published:** 2022-09-06

**Authors:** Darshankumar M Raval, Vaishnavi M Rathod, Riya K Dobariya, Milauni P Dave, Nilay S Patel

**Affiliations:** 1 Department of General Medicine, Sir Sayajirao General Hospital and Medical College Baroda, Vadodara, IND

**Keywords:** unclassified ataxia, hereditary diseases, early onset, rare autosomal recessive disorder, cerebellar-ataxia

## Abstract

Ataxia is a syndrome of imbalance and incoordination, categorized as hereditary ataxias, degenerative ataxias (non-hereditary), and acquired ataxias. Hereditary ataxia is further classified based on its mode of inheritance. Here, we have reported a case of early-onset autosomal recessive cerebellar ataxia with retained reflexes in a young male with positive family history. A young male presented with ten years history of tremors in both hands and head, aggravated with work and relieved with rest, and imbalance while walking, which has now progressed to the level where the patient cannot walk without support. The patient’s younger brother also had a similar history. Central nervous system examination revealed cerebellar ataxia with retained reflexes. After ruling out other causes of ataxia in this age group by investigations, we could make the diagnosis of early-onset cerebellar ataxia with retained tendon reflexes (autosomal recessive). Presenting as a disease of variable presentation, the important diagnostic cues are classification and localization of ataxia. The investigations should be focusing on those cases of ataxias that are treatable. Family history is important to identify hereditary ataxias, as well as in genetic counselling of the affected patients.

## Introduction

Ataxia is a disorder of imbalance and incoordination, occurring as a result of pathological abnormalities in the cerebellum, pontocerebellar pathways, spinocerebellar pathways, or afferent proprioceptive inputs (sensory ataxia) [1]. Anita Harding’s classification of ataxia (1980), based on clinical criteria, restored the interest of neurologists in ataxia and led to the emergence of various molecular tests to recognize several genetic loci leading to ataxia syndromes. Presently, ataxias can be categorized into three main groups: acquired ataxias, degenerative ataxias (non-hereditary) and hereditary ataxias [2]. Patients with ataxia of early onset (age < 25 years) and normal parents are likely to have autosomal recessive inheritance, whereas a familial disorder involving successive generations is likely to be autosomal dominant ataxia. In the case of adult-onset sporadic ataxia, making a diagnosis becomes quite difficult where a broad range of genetic and non-genetic causes are needed to be scrutinized [3]. Here, we report a case of an autosomal recessive early-onset cerebellar ataxia with retained reflexes.

## Case presentation

**Figure 1:**
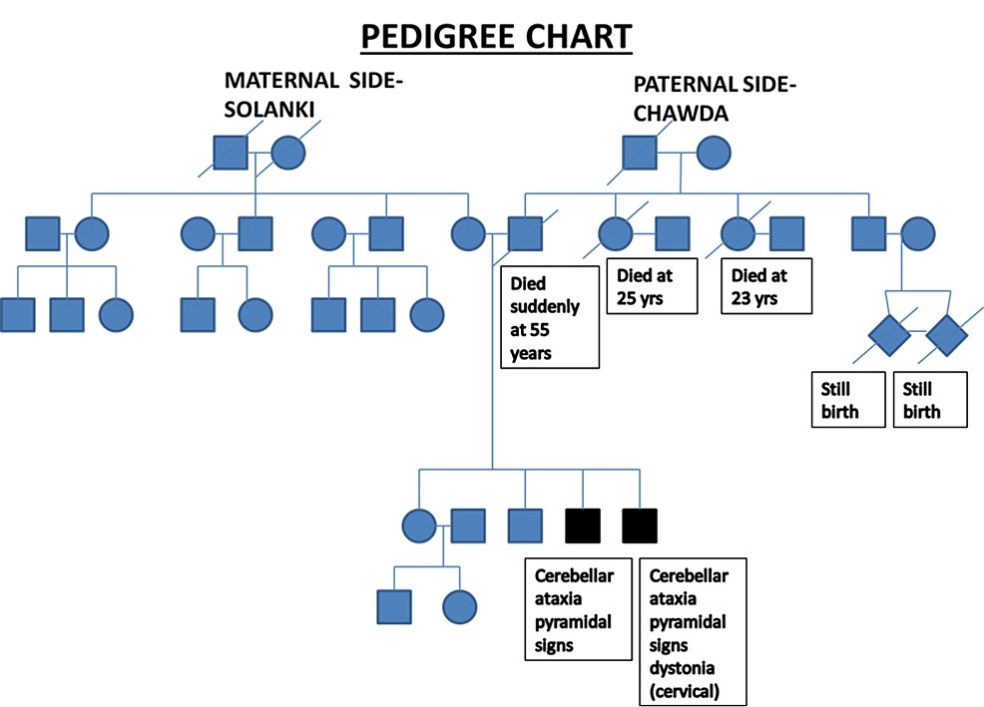
Pedigree chart of the patient's family

A 24-year-old male presented with a history of insidious onset and gradually progressive tremors of both hands and the head, with a progressive imbalance in walking for more than 10 years. The tremors of his hands were first noticed by him as a reduced speed of writing despite being able to hold the pen properly, which use to aggravated with voluntary action and relived on rest. Imbalance in walking was first noticed while walking on uneven ground which gradually progressed to repeated falls during walking even on a smooth surface, and on presenting to the hospital, he could walk with support only. He also complained of slipping slippers with knowledge. He had difficulty speaking fluently but no difficulty chewing or swallowing. There were no complaints of weakness in upper limbs, loss of sensations, bladder/bowel disturbances, or visual disturbances. There was no history suggestive of cognitive decline, seizures, or myoclonic jerks. However, similar complaints were present in his younger brother with similar progression, whereas his parents were normal (Figure 1). There was no history of consanguineous marriage in the family.

On examination, the patient was vitally stable, with normal cardiac, respiratory, and gastrointestinal system examination. Central nervous system exam revealed spastic weakness in lower limbs with impaired deep sensation in bilateral distal toes and the presence of cerebellar signs (Table 1).

**Table 1:** Thorough central nervous system examination

Examination Points	Findings
1. Higher Function	Conscious, Oriented. Normal Attention, Memory, and Praxis. Ataxic Speech.
2. Cranial Nerves	Normal with full extraocular movements.
3. Motor System	
- Nutrition	Normal in all four limbs
- Tone	Increased in bilateral lower limbs – spasticity.
-Power	Weakness of bilateral dorsiflexors in lower limbs
-Abnormal Movement	Head Titubation, Intentional tremors in both hands
4. Sensory System	
- Superficial Sensation	Normal
- Deep Sensation	Joint position and proprioceptive loss in distal toes
-Cortical Sensation	Normal
5. Reflex	
- Superficial reflexes	Normal
- Deep reflexes	Normal
6. Cerebellar Signs	
- Dyssynergia	Present
- Dysdiadochokinesia	Present
- Dysmetria (Finger-Nose test)	Present (Impaired)
- Intention Tremor	Present
- Nystagmus	Horizontal nystagmus in primary position.
- Titubation	Present
- Gait Ataxia	Present
- Speech disturbance	Scanning speech
- Knee Heel test	Impaired
7. Signs of meningeal irritation	Absent
8. Gait	Spastic Ataxic Gait
9. Peripheral nerves & vessels	Normal
10. Back & Spine	Normal

**Figure 2:**
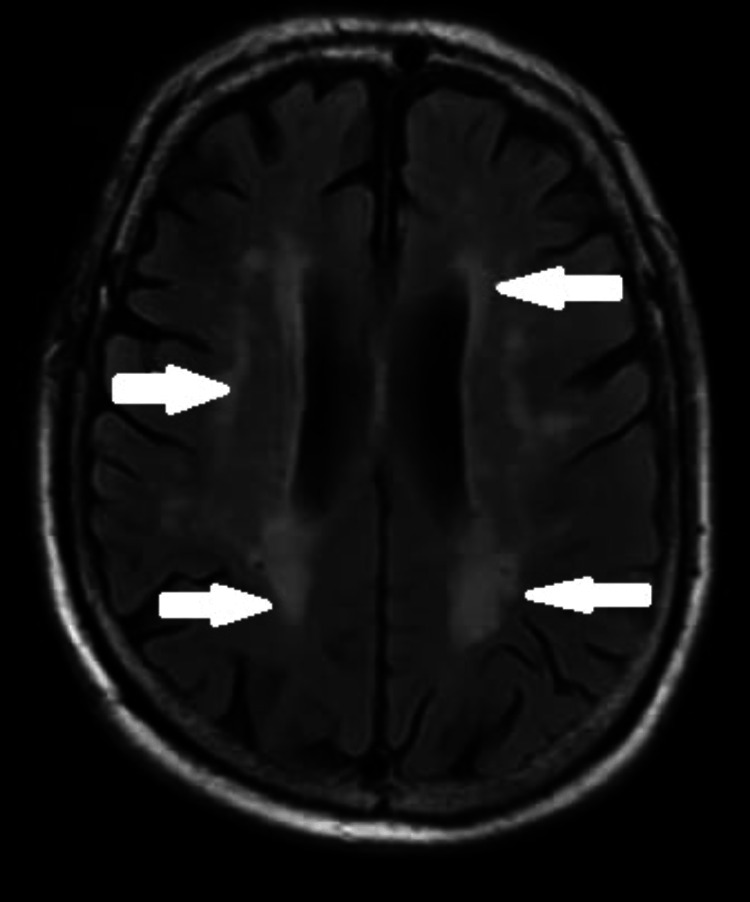
Hyperintense signal changes in the deep white matter in MRI T2 FLAIR image MRI - Magnetic resonance imaging; FLAIR - Fluid-attenuated inversion recovery

MRI brain showed T2/FLAIR (Fluid-attenuated inversion recovery) hyperintense signal changes in deep white matter (Figure 2). In addition, vitamin E levels and lipid levels were also within normal limits, along with other routine blood parameters. Electrocardiogram, echocardiography, and random blood sugars were normal, which ruled out cardiomyopathy and diabetes. Genetic testing done for GAA repeats in the frataxin gene on chromosome 9 for Friedreich’s ataxia was also negative. So after ruling out all other causes by appropriate investigations, our final impression was early-onset cerebellar ataxia with retained reflexes (autosomal recessive).

## Discussion

Sporadic cerebellar ataxias

It is an emergency both diagnostic and therapeutic, as its onset is acute (within hours to days) considering meningitis, cerebellar stroke, cerebellar abscess, vitamin B1 deficiency, and drug intoxication as probable causes. The differential diagnosis for subacute to chronic onset progressive cerebellar ataxia includes cerebellar tumor, Creutzfeldt-Jakob disease, paraneoplastic syndrome, celiac disease, Whipple’s disease, autoimmune thyroiditis, neurodegenerative diseases like multiple system atrophy, and ataxia associated with autoantibodies to glutamic acid decarboxylase [4].

Autosomal recessive cerebellar ataxias (ARCA)

One of the important ataxia in this group is Friedreich’s ataxia (FRDA) which involves corticospinal tracts, sensory peripheral nerves, dorsal root ganglia, and dentate nuclei. Patients of FRDA are having progressive early-onset ataxia involving limbs and gait, instability in ocular fixation, dysarthria, absent vibration, and proprioceptive sense as well as absent reflexes with the presence of signs of pyramidal tract lesions, without progressive cerebellar atrophy which is a major differentiating feature from other forms of ataxias. Cardiomyopathy, diabetes mellitus, scoliosis, and pes cavus can also be present [5]. The frataxin gene on chromosome 9q13 shows the expansion of GAA triplets on its first intron in most of the patients [6]. Close mimickers of FRDA are abetalipoproteinemia, ataxia with vitamin E deficiency (AVED), and Refsum’s disease [7]. Apart from ataxia, patients of AVED may have retinitis pigmentosa and cardiomyopathy. The neurological manifestations of abetalipoproteinemia are similar to AVED, however, lipid malabsorption, hypocholesterolemia, and acanthocytosis are also present. Ataxia, sensorineural deafness, peripheral polyneuropathy, retinitis pigmentosa, skeletal abnormalities, anosmia, ichthyosis, arrhythmias, cardiac myopathy, and renal failure, are features of Refsum’s disease [8]. Cases of abetalipoproteinemia and AVED have been reported from India, although without genetic confirmation [9,10].

ARCAs with earlier age of onset than FRDA and having cerebellar atrophy as a prominent feature on neuroimaging comprise ataxia telangiectasia (AT), autosomal recessive spastic ataxia of Charlevoix-Saguenay (ARSACS), AT-like disorder, ataxia with oculomotor apraxia (AOA) - type 1 and type 2, infantile-onset spinocerebellar ataxia (IOSCA), Marinesco-Sjögren’s syndrome (MSS), and Cayman ataxia (CA) [7].

Autosomal dominant cerebellar ataxias (ADCAs)

Spinocerebellar ataxias (SCAs), an autosomal dominant form, comprise a large group of heterogeneous neurodegenerative disorders, with only 40 SCAs having been described to date [11]. In most cases, symptoms appear between 30 to 50 years of age, with progressive atrophy of the cerebellum. ADCAs other than the SCA classification are neuroferritinopathy, dentatorubral-pallidoluysian atrophy (DRPLA), prion diseases, adult-onset leukodystrophies, and Alexander disease.

X-linked ataxia

Fragile X-associated tremor/ataxia syndrome (FXTAS) is a clinically heterogeneous syndrome, having tremors, followed by gait ataxia (late onset), cognitive impairment, and/or autonomic dysfunction, as predominant features. The latest study of 109 patients having late-onset cerebellar ataxia with/without tremor from North India has reported three cases of FXTAS [12].

Here, considering an early onset (<25years) of cerebellar ataxia with retained reflexes in a young male, and a significantly positive family history, we arrived at the probable diagnosis of hereditary cerebellar ataxia, most probably autosomal recessive as there is a skipping of generations ruling out autosomal dominance. X-linked and maternal inheritance was also ruled out through the pedigree chart.

There was no cerebellar atrophy in the MRI with levels of lipid profiles and vitamin E being unremarkable. Investigations to rule out other causes were also carried out. There was no associated cardiomyopathy or diabetes. Considering it to be a variant of Friedrich’s ataxia with retained reflexes, being the most common ARCA in India, genetic testing for the same was sent (GAA repeats in the frataxin gene on chromosome 9), which turned out to be negative.

Early-onset cerebellar ataxia with retained tendon reflexes

It is a clinical and genetic syndrome different from Friedreich’s ataxia, associated with dysarthria, pyramidal signs in the limbs, increased or normal upper limb reflexes and knee jerks, and sensory loss in some cases. In the majority of patients, inheritance is probably autosomal recessive in nature. The distinguishing feature from Friedreich’s ataxia is the preservation of tendon reflexes, whereas the absence of optic atrophy, diabetes mellitus, cardiomyopathy, and severe skeletal deformity are the other differentiating features from Friedreich’s ataxia. The outcome is better in such cases than that of Friedreich’s ataxia, and patients continued to be ambulatory approximately for a decade or longer.

## Conclusions

Having a variable presentation, diagnosis of ataxia is quite challenging, for which classification and localization of ataxias are used as facilitators for making the diagnosis. AVED, abetalipoproteinemia, ARCA type 2 due to coenzyme Q10 deficiency, Niemann Pick type C, and Refsum’s disease are examples of treatable ataxias, hence, efforts should always be made to investigate them. About 40% of hereditary ataxias are still unclassified. In the view of a need for genetic counselling in affected patients, family history should always be sought. As hereditary ataxias may present as a sporadic type, one must be watchful while making the diagnosis.
